# Development of Dietary Thiol Antioxidant via Reductive Modification of Whey Protein and Its Application in the Treatment of Ischemic Kidney Injury

**DOI:** 10.3390/antiox12010193

**Published:** 2023-01-13

**Authors:** Yang Sui, Rui Jiang, Manabu Niimi, Jingru Hong, Qiaojing Yan, Zhuheng Shi, Jian Yao

**Affiliations:** 1 Division of Molecular Signaling, Department of the Advanced Biomedical Research, Interdisciplinary Graduate School of Medicine, University of Yamanashi, Chuo City 409-3898, Japan; 2 Division of Molecular Pathology, Interdisciplinary Graduate School of Medicine, University of Yamanashi, Chuo City 409-3898, Japan

**Keywords:** dietary protein, reductive modified whey protein, antioxidant, sulfenic acid, ischemia reperfusion injury, hydrogen peroxide, oxidative stress

## Abstract

Thiol antioxidants play important roles in cell and body defense against oxidative stress. In body fluid, albumin is the richest source of thiol antioxidants. One recent study showed that the reductive modification of thiol residues in albumin potentiated its antioxidative activity. Given that whey protein (WP) contains albumin and other thiol-active proteins, this property of WP could be exploited to develop novel thiol antioxidants. The aim of this study was to address this possibility. WP was reductively modified with dithiothreitol (DTT). The modified protein exhibited significantly elevated free sulfhydryl groups (-SH) and thiol antioxidative activity. It detoxified H_2_O_2_ and prevented H_2_O_2_-initiated protein oxidation and cell death in a -SH group-dependent way in vitro. In addition, it reacted with GSH/GSSG and altered the GSH/GSSG ratio via thiol–disulfide exchange. In vivo, oral administration of the reductively modified WP prevented oxidative stress and renal damage in a mouse model of renal injury caused by ischemia reperfusion. It significantly improved renal function, oxidation, inflammation, and cell injury. These protective effects were not observed in the WP control and were lost after blocking the -SH groups with maleimide. Furthermore, albumin, one of the ingredients of WP, also exhibited similar protective effects when reductively modified. In conclusion, the reductive modification of thiol residues in WP transformed it into a potent thiol antioxidant that protected kidneys from ischemia reperfusion injury. Given that oxidative stress underlies many life-threatening diseases, the reductively modified dietary protein could be used for the prevention and treatment of many oxidative-stress-related conditions, such as cardiovascular diseases, cancer, and aging.

## 1. Introduction

Oxidative stress (OS) is the state of an imbalance in reactive oxygen species (ROS) generation and ROS removal, which leads to the accumulation of ROS, causing ROS-mediated cell responses and damage. OS occurs in various pathological situations and has been recognized as a common mechanism behind many life-threatening diseases [1,2]. ROS are produced from oxygen during cellular metabolism and the ROS family includes a large class of molecules with various pathophysiological activities. Among different ROS, superoxide anion (O_2_^•−^), hydrogen peroxide (H_2_O_2_), and hydroxyl radical (OH^•^) are known to be especially important because of their roles in various diseases. To counterbalance the effects of ROS, our body is equipped with multiple antioxidants, which deplete ROS, counteract ROS-induced cell responses, and protect cells against ROS-induced cell damage.

The thiol antioxidants play a vital role in the body’s defense against ROS [3]. It has multiple functions, such as radical depletion, metal chelation, and the integration of antioxidative reactions vis thiol–disulfide exchange. In addition, the redox status of intracellular and extracellular thiols also influences protein structures, enzyme activity, and transcription factor activity, thus regulating cell function and survival.

One of the most important protein thiol antioxidants in the extracellular space is albumin (Alb) [4,5]. As the most abundant plasma protein, the free sulfhydryl (-SH) group at Cys34 of Alb is the largest source of free thiol within body fluid. It detoxifies H_2_O_2_ and other oxidants in the serum. At the same time, thiol in Alb is also susceptible to oxidative modifications [5,6,7,8]. In consequence, serum Alb exists in both reduced (mercaptalbumin) and oxidized forms (non-mercaptalbumin) [9]. In reduced Alb, the reactive -SH group Cys34 is free, whereas, in oxidized Alb, it forms mixed disulfide bonds with other molecules or exists as oxidized products [5,6]. The redox status of its cysteine residues in Alb reflects the systemic oxidative condition and has been used as an indicator of oxidative stress in clinical studies [8,10]. Recently, we have successfully converted normal albumin into a potent thiol antioxidant via freeing the -SH groups of cysteines in Alb [11]. This experience prompted us to speculate that the same strategy could also be used to obtain cysteine-rich dietary proteins.

Milk is one of the primary sources of protein in the diet. It contains multiple nutrients with various biological activities and has many health-promoting effects. It is one of the most extensively used dietary products [12,13]. Whey protein (WP) is a by-product of the cheese-making process, which contains approximately 20% of the total protein content in bovine milk. The major components of WP include β-lactoglobulin, α-lactalbumin, immunoglobins, serum albumin, lactoferrin, and lactoperoxidase. Structurally, most proteins contain multiple cysteine residues, which form structural disulfide bonds or exist as a free -SH group [14,15]. Against this background, WP could be an ideal candidate to test whether the reductive modification of cysteines in WP could provide it with antioxidative activity. The purpose of this study was to address this possibility.

Renal ischemia reperfusion injury (IRI) is caused by a temporary loss of blood flow to the kidneys, followed by the recovery of the flow and reoxygenation. It is one of the major causes of acute kidney injury (AKI), contributing to high mortality caused by several clinical situations, such as infarction, sepsis, and renal transplantation. Currently, the pathogenesis of IRI is still incompletely understood. It is generally accepted that, during the phase of ischemia and subsequent reperfusion, the generation of reactive oxygen species (ROS) initiates a series of harmful cellular reactions, leading to inflammation, cell injury, and loss of kidney function. Antioxidative therapies against ROS are effective in the treatment of IRI [16,17]. However, in clinical practice, effective antioxidative regimens are still limited [18]. It is highly desirable to acquire a better understanding of the mechanisms and develop more effective therapeutic approaches. 

In this context, we, therefore, tested whether the reductive modification of cysteines in WP could convert it into a protein thiol antioxidant and whether the modified dietary protein could prevent renal IRI. Here, we present our results, showing that reductively modified whey protein (R-WP) displayed strong antioxidative activity, which could be used to prevent and treat renal IRI via oral administration.

## 2. Materials and Methods

### 2.1. Materials

Bovine serum albumin (BSA, Faction V) was purchased from Iwai Chemical Company (Tokyo, Japan). Dimedone was bought from Tokyo Chemical Industry (Tokyo, Japan), and Alexa 680 Fluor C2 maleimide was from Thermo Scientific (Rockford, IL, USA). Anti-cysteine sulfenic acid antibody was obtained from Millipore (Burlington, MA, USA). HRP-conjugated anti-rabbit or mouse IgG, anti-β-actin, and anti-caspase 3 antibodies were purchased from Cell Signaling, Inc. (Beverly, MA, USA). A blood urea nitrogen (BUN) kit was purchased from Thermo Fisher Scientific (Frederick, MD, USA). Creatinine was from Cayman Chemical Company (Ann Arbor, MI, USA). Anti-NGAL antibody was obtained from Adipogen (San Diego, MI, USA). Maleimide and all other chemicals were bought from Sigma-Aldrich (St. Louis, MO, USA).

### 2.2. Whey Protein and Its Preparation

The low-temperature-processed whey protein powder was kindly gifted by Asama Chemical Co. Ltd. (Tokyo, Japan) [19,20,21]. The powder was dissolved in distilled water and used for the reductive modification and other experiments.

### 2.3. Mice

Adult male or female C57BL/6 mice (20~30 g) were housed in a temperature-controlled facility with a 12-h light cycle and fed a standard laboratory diet. The animal study was approved by the Animal Care and Use Committee of Yamanashi University and carried out according to animal experimentation guidelines and rules.

### 2.4. Induction of Renal Ischemia Reperfusion Injury

The mouse model of renal IRI was performed as previously reported [22]. The mice were randomly allocated to the sham-operated (control), renal ischemia reperfusion (I/R), and renal I/R treated with differently modified WP groups. Mice were anesthetized by intraperitoneal injection of sodium pentobarbital (40 mg/kg) (somnopentyl^®^, Kyoritsu seiyaku corp., Tokyo, Japan) and placed on a 38 °C heating pad. The abdomen was shaved and cleaned with 70% ethanol. A midline incision was made. The bowel was moved to reveal renal hila. Each renal pedicle (artery, vein, and ureter) was clamped for 40 min with clips, followed by removing the clips to recover the normal blood flow. Sham animals underwent the same procedure without pedicle clamping. Twenty-four hours after the operation, mice were anesthetized to collect blood and tissue samples.

### 2.5. Western Blot Analysis

Western blot was performed as previously described [11]. Cell lysates were made using 1× SDS lysis buffer (62.5 mM Tris-HCl, 2% SDS, 10% glycerol), and tissue samples were homogenized in RIPA lysis buffer together with a proteinase inhibitor cocktail. The protein concentrations were measured with the Micro BCA Protein Assay Kit (Thermo Fisher Scientific, Waltham, MA, USA). An equal amount of protein was loaded onto 10% SDS-PAGE, separated, and transferred to PVDF membranes with wet-blotting apparatus. The membrane was blocked with either 5% skimmed milk or 3% BSA in 0.1% Tween-20 PBS solution (TPBS) for at least 1 h, followed by incubation with the primary antibody at an appropriate dilution at 4 °C overnight. After washing with TPBS, the membrane was allowed to react with peroxidase-conjugated secondary antibody for 1 h and detected for the bands with the enhanced chemiluminescence system (Nacalai Tesque, Kyoto, Japan). The chemiluminescent signal was captured with a Fujifilm image LAS-1000 analyzer (Fujifilm, Tokyo, Japan), and the intensity of the bands was quantified with the NIH Image J software (http://rsb.info.nih.gov/ij, accessed on 10 January 2022). β-actin or EZ blue staining was used to ensure equal loading of sample proteins.

### 2.6. Modification of Whey Protein and Albumin with Reductive Chemical DTT

WP at 120 mg/mL in distilled water was incubated with 100 mM DTT overnight at 4 °C, which was followed by dialysis against a large amount of distilled water or saline (0.9% sodium chloride in distilled water) in dialysis tubing (3500 MWCO, Thermo Scientific, Rockford, IL, USA) with more than 5 changes of dialysis solution. A portion of the modified protein was further treated with 50 mM maleimide at 4 °C overnight to block the -SH groups, followed by the dialysis procedure to remove the remaining small molecules and metabolites. Finally, the modified proteins were assayed for concentrations, examined for changes in the amount of -SH groups, and stored at −80 °C for subsequent experiments.

### 2.7. Maleimide-Labeling Assay

This assay was performed as we have previously described [11]. Differently modified WPs with or without additional treatment were incubated with Alexa Fluor 680 C2 maleimide (red fluorescence at the final concentration of 5 μM) at 4 °C for 2 h. After the interaction, the protein samples were subjected to SDS-PAGE separation, and the fluorescent signal in the gel was captured with a Fujifilm image LAS-1000 analyzer (Fujifilm, Tokyo, Japan). The intensity of the band was quantified with ImageJ software. EZ blue staining of the gel was performed to confirm the equal loading of proteins.

### 2.8. Measurement of Free Sulfhydryl Groups (-SH) in Proteins Using Ellman’s Reagent

Free thiol concentrations in differently modified WPs were measured following the protocol provided by Thermo Scientific with minor modifications, as we have reported [11]. Briefly, cysteine standard and samples dissolved in 0.1 M Tris buffer at pH 8.0 were allowed to react with Ellman’s reagent (Dojindo Molecular Technologies Inc., Rockville, MD, USA) for 15 min. Absorbance was measured at 412 nm. The concentration of free -SH in samples was calculated based on the standard curves generated from L-cysteine and expressed as μmol/g WP.

### 2.9. Enzyme-Linked Immunosorbent Assay and Renal Function Analysis

Forty-milligram renal cortical tissues were homogenized in 200 μL RIPA lysis buffer. The supernatants were collected and assayed for IL-1α and TNF-α using an Enzyme-Linked Immunosorbent Assay (ELISA) Kit from Peprotech, following the manufacturer’s instructions (Rocky Hill, NJ, USA).

### 2.10. Kidney Histology Analysis

Kidney histology was performed as previously reported [11]. Kidney tissues were fixed with 10% formalin, embedded in paraffin, sliced into 4-μm sections, and stained with a standard hematoxylin and eosin (H&E) procedure.

### 2.11. Detection of Sulfenic Acid

The protein level of sulfenic acid (-SOH) formation was determined as previously reported by Saurin et al. [23]. Briefly, serum at the dilution of 20 to 50 folds or cellular and tissue lysates at the concentration of 1~5 mg/mL were allowed to react with 1 mM dimedone at RT for 20 min. After mixing with a fivefold non-reducing sample buffer, the samples were subjected to Western blot analysis using an anti-dimedone antibody.

### 2.12. Cell Culture

For cell passage and expansion, normal renal tubular epithelial cell line NRK cells (ATCC, Rockville, MD, USA) were cultured with DMEM/F12 containing 5% FBS and 1% antibiotic and antimycotic solution in a humidified atmosphere of 5% CO_2_/95% air at 37 °C. For experiments, cells were seeded into culture plates in a culture medium containing 0.5% FBS and stimulated as described in the figure legend.

### 2.13. Calcein-AM and Propidium Iodide (PI) Staining

Calcein-AM/PI live/dead staining was performed using an assay kit from Dojindo, Kumamoto, Japan, following the protocol provided by the manufacturer. Briefly, a mixture of Calcein-AM (green) and PI (red) solution was added into culture wells and allowed to react for 10~20 min. Then, the fluorescent images of cells were observed and photographed under a fluorescent microscope. Green cells (positively stained with Calcein-AM) were considered alive, while red cells (stained with PI) were deemed to be dead.

### 2.14. Assessment of Cell Viability with WST Reagent

Cells grown in 96-well culture plates were stimulated with various chemicals for the indicated period and exposed to WST reagent (Dojindo, Kumamoto, Japan) for 60 min. The optical density (OD) was measured with a spectrometer at the wavelength of 450 nm.

### 2.15. Analysis of Hydrogen-Peroxide-Quenching Capacity of the Modified WP

The quenching capacity of the modified WP on H_2_O_2_ was assessed by determining the changes in H_2_O_2_ concentrations before and after the reaction with differently modified WPs using an H_2_O_2_ assay kit from Cayman Chemical Company (Ann Arbor, MI, USA, 600050). Briefly, H_2_O_2_ at the concentration of 1 mM was allowed to react with or without 180 μg of the differently modified WPs for 1 h. Next, an aliquot of the solution (40 μL) was mixed with the same volume of assay buffer and enzyme reaction solution, and then incubated for an additional 30 min in the dark at RT, followed by fluorescence measurement at a 530 nm excitation wavelength and emission at a 590 nm wavelength with a SpectraMax^®^ GEMINI EM Microplate Reader.

### 2.16. GSH/GSSG Assay

GSH and GSSG were measured using an assay kit from Dojindo (Product code: G257; Dojindo Molecular Technologies, Inc; Kumamoto, Japan), as we have previously reported [11]. Briefly, two sets of samples were prepared in 0.05% SSA solution to determine GSH and GSSG, respectively. After incubation with the buffer solution at 37 °C for 1 h, the samples were allowed to react with the coenzyme and substrate working solution for an additional 30 min. The absorbance of the developed color was read with a spectrometer at 405 nm. The concentration was calculated based on the standard curve obtained from GSH and GSSG controls.

### 2.17. Measurement of BUN, Creatinine

Blood urea nitrogen (BUN) and creatinine were measured with commercial kits, following the protocols provided by the manufacturers. Briefly, sera with or without dilution were allowed to react with assay buffer for the required time. The optical absorption was measured at the wavelength of 450 nm for BUN and 490 nm for creatinine with a spectrophotometer (SpectraMax 340, Molecular Devices, Tokyo, Japan).

### 2.18. Statistical Analysis

Data are expressed as mean ± SE. Comparison of the two groups was performed using Student’s *t*-test. For multiple comparisons with the same control, one-way ANOVA analysis and post-hoc comparisons (Dunnett’s test and Tukey’s test) were made. All the analyses were conducted using Microsoft Excel (Microsoft, Redmond, WA, USA) or GraphPad Prism8 software (Version 8, GraphPad, San Diego, USA). *p* < 0.05 was considered statistically significant.

## 3. Results

### 3.1. Reductive Modification of WP Increases Its -SH Activity

**Figure 1:**
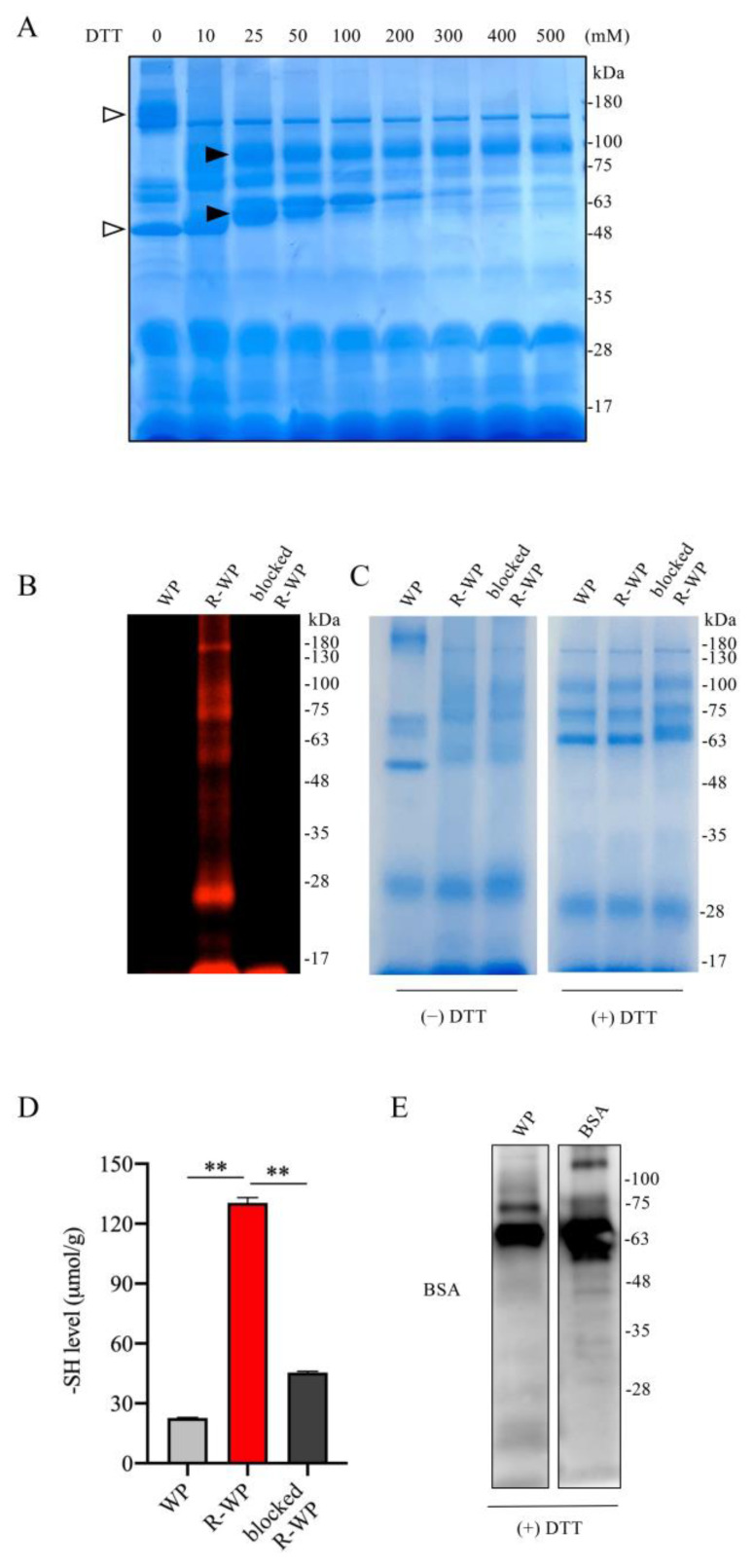
Reductive modification of WPs with DTT leads to an increased number of -SH groups. (**A**) Effect of DTT treatment on WP structure. WP at the concentration of 120 mg/mL was treated with the indicated concentrations of DTT for 30 min at RT. A portion of the treated samples were centrifuged, loaded into 10% SDS-PAGE, separated, and stained with EZ blue. Note the shift in bands after DTT treatment (arrow). (**B**–**D**) Increased number of -SH groups in R-WP. WP at 120 mg/mL was allowed to react with or without 100 mM DTT overnight at 4 °C. After removing the remaining DTT and other small-molecular metabolites via dialysis, the samples were analyzed for -SH groups using the fluorescence-labeled maleimide probe. Note the markedly increased fluorescence in R-WP that could be blocked entirely with unlabeled maleimide (**B**). To confirm the equal loading of the samples, gels were stained with EZ blue (right image). Because the shift in the bands affected the judgment, the same amounts of the samples were also treated with DTT to confirm the equal loading. (**D**) Assessment of -SH concentration with Ellman’s reagent. Differently modified WPs were measured for -SH concentration with Ellman’s reagent. The concentration was calculated based on the standard curved generated from cysteine, and the data are expressed as μmol/g (*n* = 4; ** *p* < 0.01 vs. WP). (**E**) Presence of BSA in WP. Western blot analysis of WP ingredients with an anti-BSA antibody. Note the presence of a positive band located at the exact position of the BSA control.

To test whether WP could be developed as a thiol antioxidant, we used WP that was processed with low-temperature pasteurization, which led to minor protein denaturation [19,20,21]. We first examined the components of WP and the changes in structure after reductive treatment with DTT using SDS gel separation and gel staining. Figure 1A shows that incubation of WP with an increased concentration of DTT caused a downshift of several bands, suggesting the possible cleavage of the interchain disulfide bands. This effect of DTT was detectable at a wide range of concentrations, starting from 10 mM to 200 mM. Because the higher concentration of DTT caused protein precipitation (data not shown), we chose 100 mM DTT for subsequent reductive modification. The number and activity of -SH groups after the modification were confirmed with maleimide labeling and Ellman’s assay. Figure 1B,C show that DTT modification markedly increased the binding of proteins to the -SH-reactive fluorescent maleimide and -SH activity as indicated by Ellman’s assay. This effect could be entirely abolished by the pretreatment of R-WP with unlabeled maleimide. These observations indicate that reductive treatment increased -SH groups in WP.

As a component of WP [12,13], albumin has been reported to have antioxidative action [11]. We, therefore, confirmed the existence of BSA in the WP. Using a specific anti-BSA antibody, we detected a band at the location of the BSA control. Collectively, we successfully increased the -SH groups in WP via reductive modification with DTT.

### 3.2. R-WP Reacts with H_2_O_2_ and GSH/GSSG via Thiol–Disulfide Exchange

**Figure 2:**
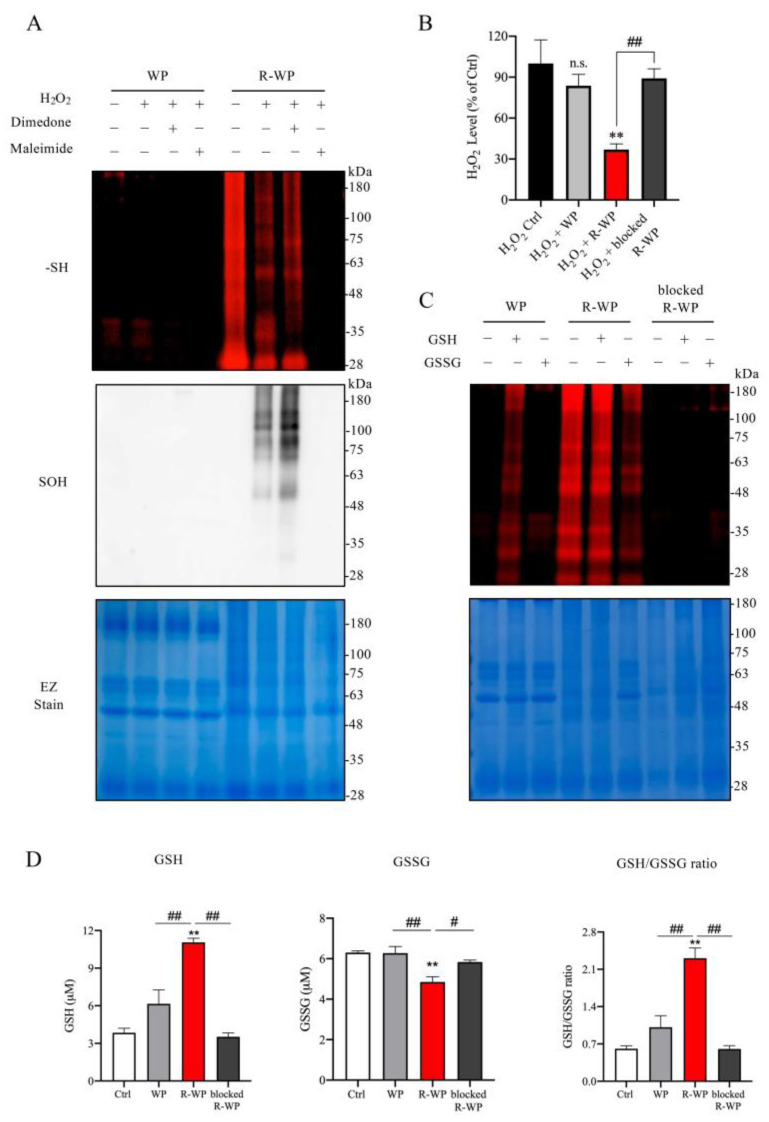
R-WP interacts with anti- and pro-oxidant H_2_O_2_ /GSH/GSSG via thiol-mediated reaction. (**A**) Interaction between differently modified WPs and H_2_O_2_. Two mg/mL normal and R-WP were pretreated with or without 1 mM dimedone or maleimide for 30 min, followed by exposure to 1 mM H_2_O_2_ for an additional 1 h at RT. Afterward, an aliquot of the reaction solution was assayed for -SH (upper panel) or -SOH groups (middle panel) using the method described in the Materials and Methods. The equal loading of samples was confirmed by staining the gel with EZ blue (lower panel). (**B**) Effect of differently modified WP on H_2_O_2_ concentration. H_2_O_2_ at the concentration of 1 mM was incubated with control distilled water or 2 mg/mL differently modified WP for 60 min at RT. Afterward, H_2_O_2_ concentration was assayed with an assay kit from Cayman. The data are expressed as a percentage relative to the control (mean ± SE; *n* = 3; n.s.: not significant; ** *p* < 0.01 vs. Ctrl, ^##^ *p* < 0.01 vs. R-WP). (**C**) Interaction between WP and GSH/GSSG. Differently modified WPs at 2 mg/mL were allowed to react with 2 mM GSH or GSSG overnight at 4 °C. After removing the unreacted GSH and GSSG through TCA/acetone precipitation, equal amounts of samples were used to determine -SH groups with the fluorescent labelled maleimide probe using the method described in the Materials and Methods. Note the altered fluorescent intensity of bands in proteins after treatment with or without GSH/GSSG. (**D**) Effect of differently modified WPs on GSH and GSSG ratio. A mixture of GSH and GSSG was allowed to react with differently modified WPs (0.1 mg/mL) for 60 min and assayed for GSH- and GSSG-like activity. Data shown are mean ± SE (*n* = 4; ** *p* < 0.01 vs. Ctrl; ^#^ *p* < 0.05, ^##^ *p* < 0.01 vs. R-WP group).

To determine whether the increased -SH groups in WP could have antioxidative action, we examined whether there existed a direct interaction between the modified protein and the major oxidant H_2_O_2_ in vitro. Figure 2A shows that incubation of the modified protein with H_2_O_2_ decreased the amount of free -SH groups in R-WP, as revealed by the reduced maleimide labeling. Conversely, the reaction markedly increased -SOH formation. This effect of the modified protein was lost after pretreatment of the protein with the unlabeled maleimide, suggesting that the interaction was mediated via -SH groups. Of note, to prevent the disulfide formation caused by H_2_O_2_, which affects the detection of -SOH, we also included a group in which the -SOH-reactive chemical dimedone was added. This treatment partially increased the detected level of -SOH, as compared with the untreated group, implying that there existed disulfide bond reformation under H_2_O_2_ exposure.

We also examined the in vitro effect of R-WP on H_2_O_2_ levels. To this end, H_2_O_2_ was exposed to the modified proteins for 60 min, and the changes in H_2_O_2_ concentration were detected. Figure 2B shows that treatment of H_2_O_2_ with R-WP caused a significant reduction in H_2_O_2_ concentration, which was abolished through the blockade of -SH groups with maleimide.

Because the ratio of GSH and GSSG is used as an indicator to evaluate the in vivo redox state [24,25], we tested whether the modified protein could directly interact with GSH/GSSH, thus altering the GSH/GSSG ratio in vitro. For this purpose, differently modified WPs were allowed to react with GSH or GSSG. The changes in GSH, GSSG, and their ratio were determined. Figure 2D shows that the incubation of R-WP with the GSH and GSSG mixture increased the GSH activity and reduced the GSSG activity. Consequently, the ratio of GSH/GSSG was elevated. This effect of R-WP was also lost when the -SH groups were blocked with maleimide, suggesting the involvement of -SH groups in the interaction. Collectively, the above observations indicate the existence of thiol-mediated reciprocal regulation among the modified WP, H_2_O_2_, GSH, and GSSG.

### 3.3. R-WP Protects Renal Tubular Cells from H_2_O_2_-Elicited Cell Injury

**Figure 3:**
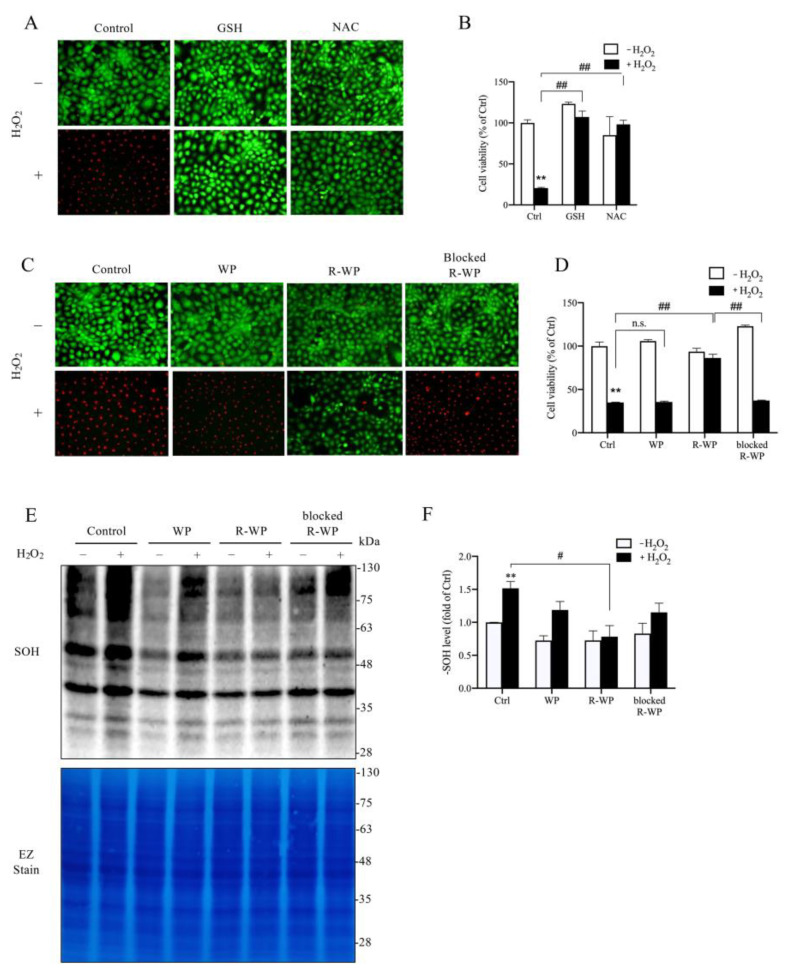
R-WP prevents H_2_O_2_-induced oxidative cell injury in cultured renal tubular epithelial cells. (**A**–**D**) Effect of small thiol antioxidants and differently modified WPs on H_2_O_2_-initiated cell injury. H_2_O_2_ at the concentration of 500 μM was incubated with 1 mM GSH, NAC, or 5 mg/mL WPs at RT for 30 min. After centrifugation, the supernatants were added to cultured cells and the cell viability at 6 h was determined using Calcein AM/PI staining (**A**,**C**) and formazan formation (**B**,**D**). Note the red PI-positive dead cells in A and C and their prevention by GSH, NAC and R-WP protein. The data in B and D are mean ± SE (*n* = 4; n.s.: not significant; ** *p* < 0.01 vs. Ctrl; ^##^ *p* < 0.01 vs. R-WP group). (**E**,**F**) Effect of differently modified WPs on H_2_O_2_-induced protein oxidation. Cells were treated similarly as above for 1.5 h. The cellular proteins were assayed for -SOH formation (**E**). The equal loading was confirmed by EZ staining. The densitometric quantitation of the blot in (**E**) is shown in (**F**). The results are expressed as the fold change against the control. Data shown are mean ± SE (*n* = 3; ** *p* < 0.01 vs. Ctrl; ^#^ *p* < 0.05 vs. R-WP group).

We then moved on to test whether the R-WP protein could protect cultured cells from oxidative injury. For this purpose, we exposed renal tubular epithelial NRK-52E cells to H_2_O_2_. Figure 3A–D show that H_2_O_2_ induced NRK cell injury, as indicated by the increased number of PI-positive dead cells (red; Figure 3A,C) and the decreased formazan formation (Figure 3B,D). This cytotoxic effect of H_2_O_2_ could be prevented entirely by thiol antioxidant NAC and GSH. Intriguingly, the R-WP protein also significantly inhibited H_2_O_2_-induced cell injury through a -SH-dependent mechanism. 

Further analysis revealed that, consistent with the reduced cytotoxicity, the cellular protein oxidation induced by H_2_O_2_ was also significantly suppressed by R-WP, but not the control and -SH-blocked WP.

### 3.4. R-WP Protects Kidney from I/R-Induced Injury

**Figure 4:**
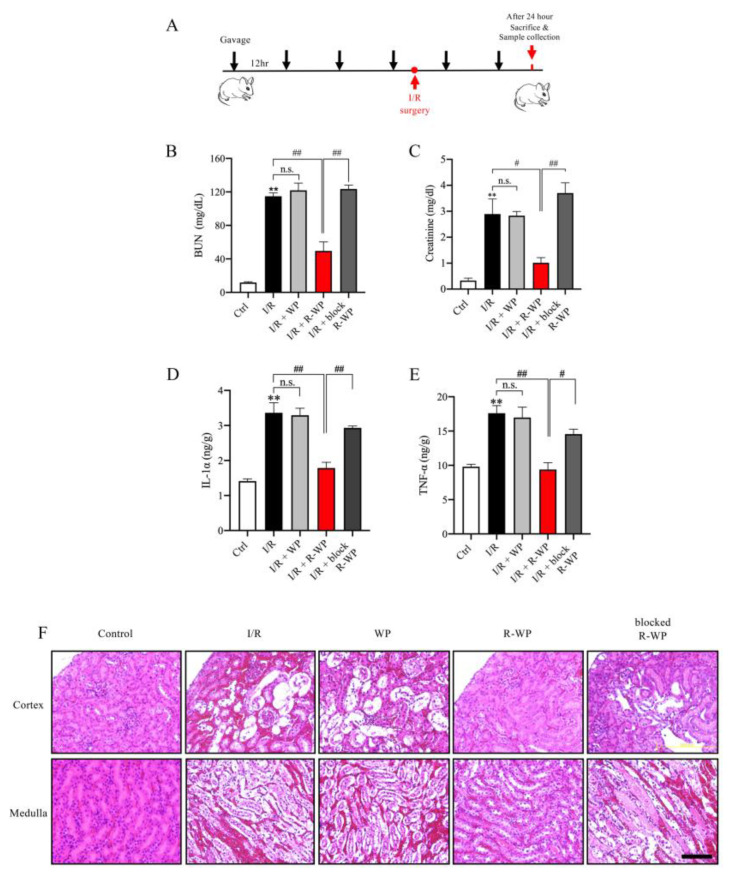
R-whey ameliorates I/R-induced changes in renal function, inflammation, and structure. (**A**) Schematic depiction of the experimental design. (**B**–**E**) Effect of WP treatment on renal function and inflammation. The blood collected from differently treated groups was assayed for BUN, creatinine, and inflammatory mediators. Data shown are mean ± SE (*n* = 4; n.s.: not significant; ** *p* < 0.01 vs. Ctrl; ^#^ *p* < 0.05; ^##^ *p* < 0.01 vs. R-WP group). (**F**) Effects of WP treatment on the renal structure. Representative images of HE staining of the renal section are shown. Note that I/R induced noticeable changes in renal tubular dilation, the disappearance of the tubular brush board, and the detachment of renal tubular cells in control and its prevention by R-WP. Scale bar, 10 μm.

To address whether the modified protein could also exert antioxidative action in vivo, we used an oxidative-centered model of renal injury induced by ischemia reperfusion (I/R) [16,17]. To this end, mice were pretreated with different forms of WP via gavage before the induction of I/R injury, and the changes in renal function, structure, and oxidative status were monitored (Figure 4A). Figure 4B–E show that I/R mice exhibited a marked increase in BUN, creatinine, as well as cytokines (IL-1α and TNF-α). R-WP treatment significantly inhibited these changes in a -SH group-dependent way. Histochemical staining revealed extensive renal tubular cell injury, as evidenced by tubular dilation, loss of tubular brush borders, and the detachment of tubular epithelial cells, which was also largely prevented by R-WP. 

**Figure 5:**
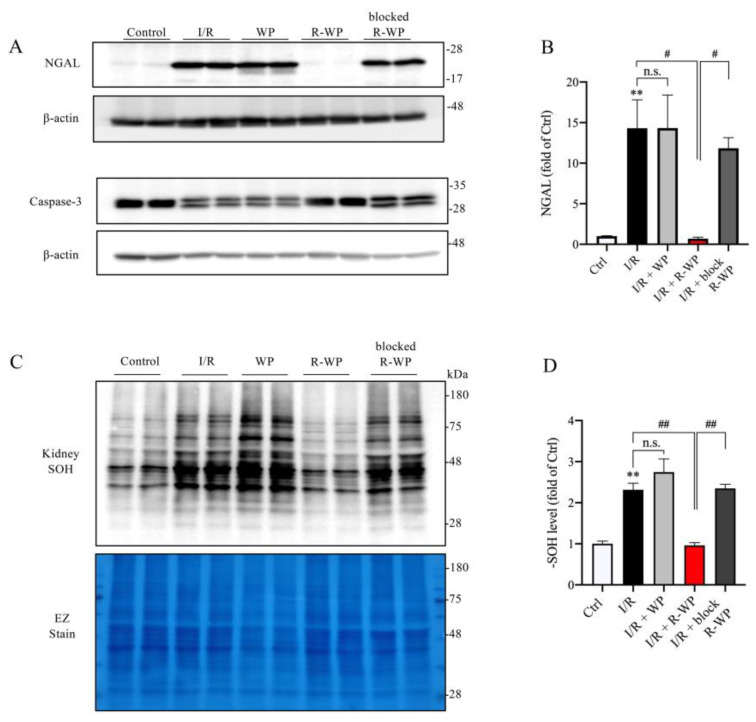
R-WP ameliorates I/R-induced renal cell injury and protein oxidation. (**A**,**B**) Effect of WP treatment on renal injury markers. Kidney lysates extracted 24 h after I/R were assayed for renal tubular cell injury marker Lipocalin-2 (NGAL) and apoptosis marker caspase-3. Note the obviously increased level of NGAL, as well as the split of caspase-3 in I/R control and its prevention by R-WP. The quantitation of the bands of NGAL is shown in (**B**). Data are expressed at fold change relative to sham control. Data shown are mean ± SE (*n* = 4; n.s.: not significant; ** *p* < 0.01 vs. Ctrl; ^#^ *p* < 0.05 vs. R-WP group). (**C**,**D**) Effect of WP treatment on -SOH formation. An equal amount of kidney lysate was treated with dimedone and subjected to Western blot analysis for -SOH level. EZ staining of the membrane was used to verify the equal loading of protein in each lane. The densitometric quantification of the signals in (**C**) is shown in bar graph in (**D**). Data shown are mean ± SE (*n* = 4; n.s.: not significant; ** *p* < 0.01 vs. Ctrl; ^##^ *p* < 0.01 vs. R-WP group).

In agreement with the histopathological findings, Western blot analysis revealed that R-WP treatment also potently prevented the appearance of the renal tubular injury marker NGAL in I/R mice. In addition, R-WP also inhibited caspase-3 activation, based on the pattern of caspase-3 in the Western blot. In control mice, caspase-3 was exhibited as one band; however, it appeared as double bands in I/R mice. This alteration was entirely abolished in mice treated with R-WP. In consistency with the changes in renal injury markers, renal protein oxidation, as indicated by the -SOH level, was also potently suppressed by R-WP (Figure 5C,D). Collectively, these observations indicate that R-WP prevents I/R-induced renal cell oxidation and injury.

### 3.5. Administration of R-WP Leads to an Improvement in Systemic Redox Status

**Figure 6:**
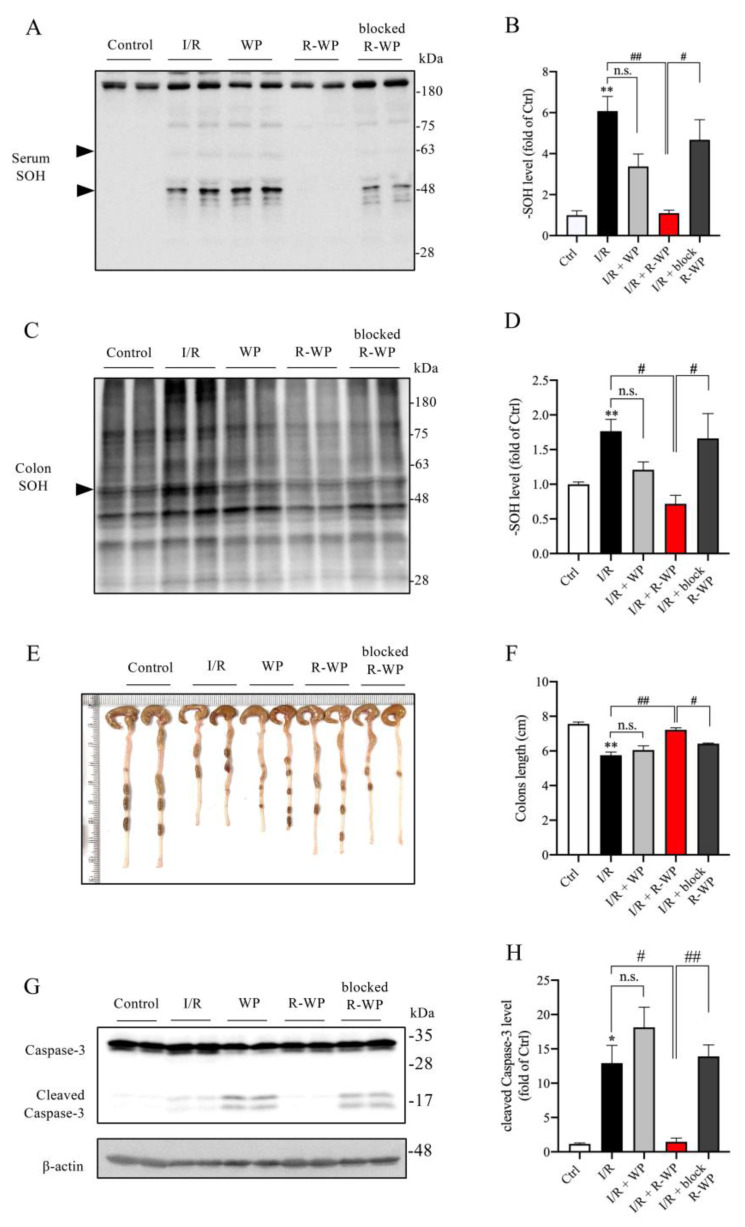
R-WP improves systemic oxidative status in renal I/R mice. (**A**–**D**) Effect of R-WP treatment on -SOH formation in serum and colon. The serum and colon lysates were assayed for the level of -SOH formation using Western blot, as described in Materials and Methods. The densitometric quantification of the bands (arrowheads in (**A**) and whole blot in (**C**)) was performed, and the results are presented as a bar graph in (**B**,**D**). Data are expressed as mean ± SE (*n* = 4; n.s.: not significant; ** *p* < 0.01 vs. Ctrl; ^#^ *p* < 0.05, ^##^ *p* < 0.01 vs. R-WP group). (**E**,**F**) Effect of R-WP treatment on the colon length. Colon lengths at 24 h after the operation were photographed, measured, and presented as a bar graph in (**F**). Data shown are mean ± SE (*n* = 4; n.s.: not significant; ** *p* < 0.01 vs. Ctrl; ^#^ *p* < 0.05, ^##^ *p* < 0.01 vs. R-WP group). (**G**,**H**) Effect of R-WP treatment on caspase-3 cleavage in colon tissues. The colon lysates were subjected to Western blot analysis for caspase-3. Note the appearance of cleaved bands at the molecular level around 17 kDa and its prevention by R-WP treatment. Data shown are mean ± SE (*n* = 4; n.s.: not significant; * *p* < 0.05 vs. Ctrl; ^#^ *p* < 0.05, ^##^ *p* < 0.01 vs. R-WP group).

Apart from the changes in the local kidney, we also examined the effect of R-WP on the systemic oxidative status. Figure 6 shows that the protein oxidation in the serum and colon was elevated in I/R mice (A–D), together with a significantly shortened colon length as well as colon cell injury, as revealed by caspase-3 cleavage (E–H). R-WP treatment, however, prevented all these changes. These observations suggest that R-WP administration improves the systemic oxidative status and attenuates I/R-induced intestinal injury.

### 3.6. Reductively Modified Albumin Similarly Alleviates Renal IRI

**Figure 7:**
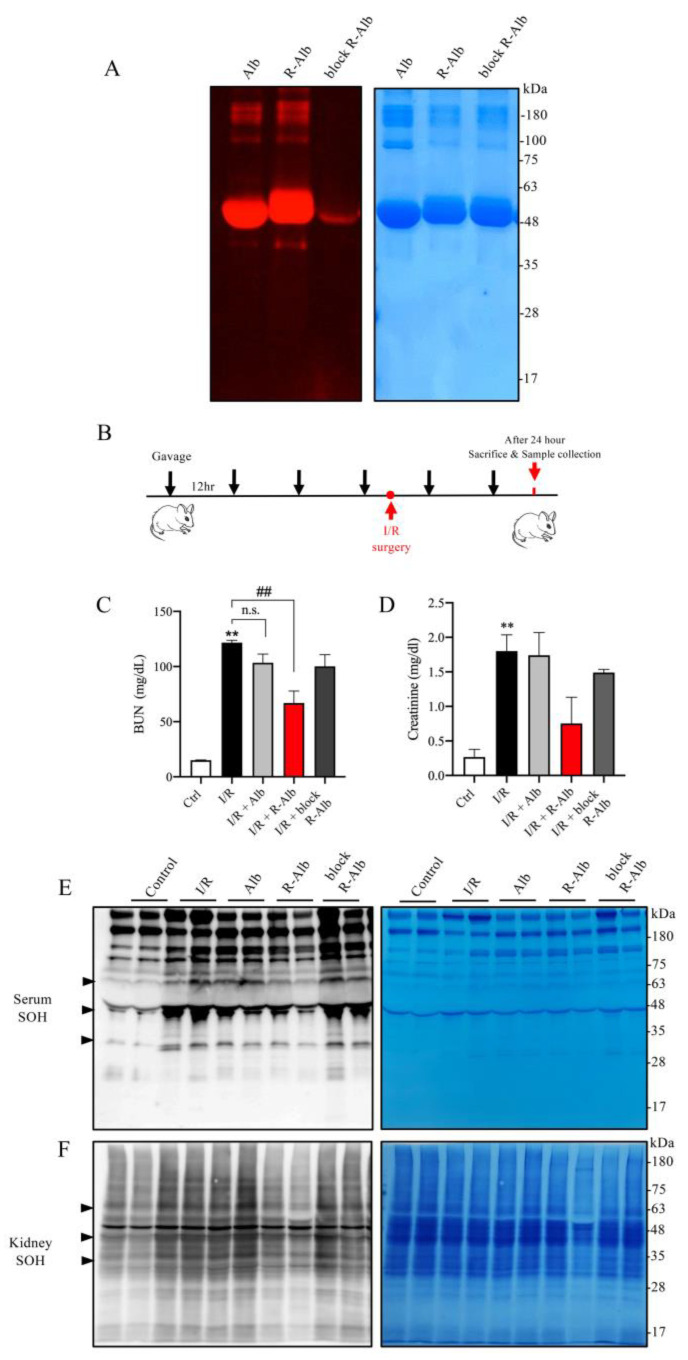
Reductively modified albumin attenuated renal I/R injury. (**A**) The influence of reductive modification on the -SH level in albumin. Albumin was reductively modified similarly to WP. After the modification, the changes in -SH groups were determined with a maleimide-based thiol fluorescent probe. EZ blue staining was performed to confirm the equal loading of protein. (**B**) The schematic depiction of the animal experiment design. (**C**,**D**) Effect of reductively modified albumin on renal function. Sera from the differently treated groups were assayed for BUN and creatinine. The results are mean ± SE (*n* = 3; n.s.: not significant; ** *p* < 0.01 vs Ctrl; ^##^ *p* < 0.01 vs. R-WP group). (**E**,**F**) The effect of R-Alb administration on protein oxidation. Serum and renal lysates were assayed for -SOH formation. The equal loading of proteins was verified with EZ blue staining. Note the obviously changed bands between the treated and untreated groups (arrowhead).

Given that albumin is a well-known component of WP, we, therefore, asked whether albumin could also attenuate renal IRI. For this purpose, we made reductively modified albumin using the same procedure as for WP. Using thiol-binding maleimide as a probe, we confirmed that the -SH activity was indeed elevated (Figure 7A). We then tested its effect on renal IRI (Figure 7B). Figure 7C,D show that the administration of reductively modified albumin (R-Alb) significantly improved renal function, as indicated by the reduced levels of BUN and creatinine. This effect of the modified albumin was associated with a marked decrease in serum and renal protein oxidation (Figure 7E,F). These observations indicate that albumin, as an ingredient of WP, may contribute to the protective actions of the reductively modified WP.

## 4. Discussion

**Figure 8:**
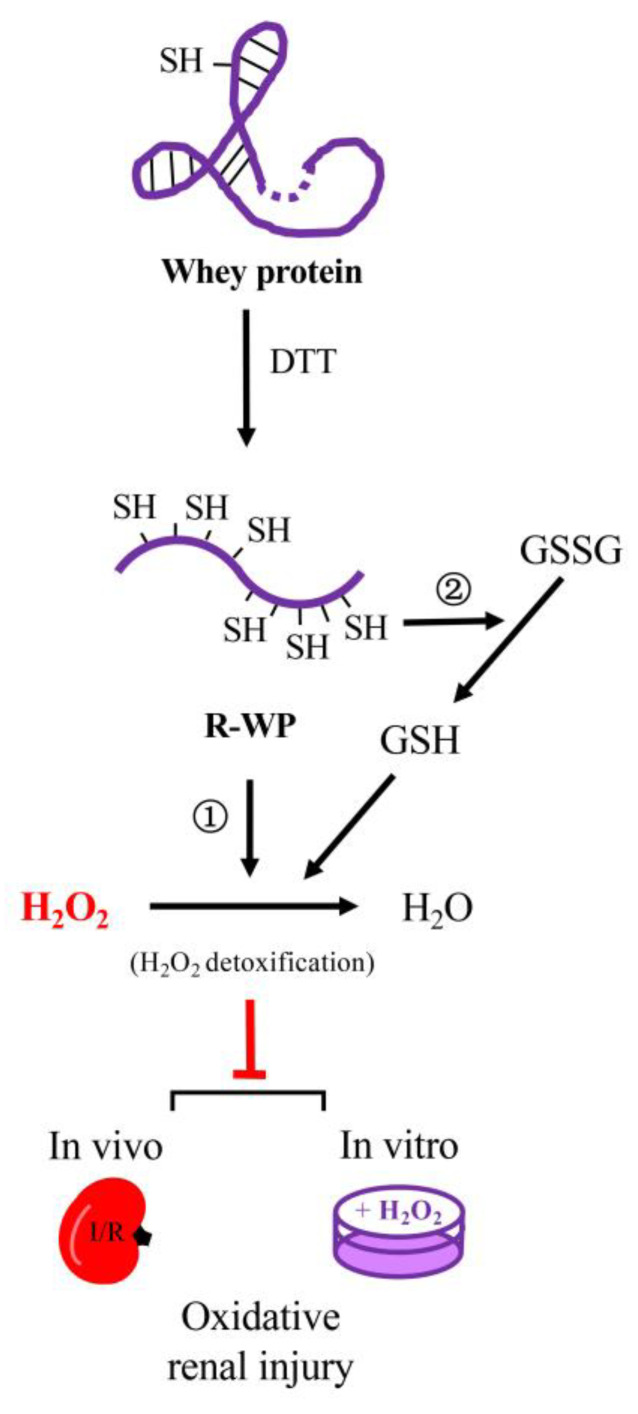
Schematic depiction of the mechanisms involved in the antioxidative actions of the reductively modified whey protein. Reductive modification of WP with DTT results in the release of -SH groups via splitting of disulfide bonds in protein structure. The exposed -SH groups exert antioxidative actions through ① direct detoxification of H_2_O_2_, and ② integration to thiol antioxidative system, consequently, leading to prevention of H_2_O_2_-induced oxidative cell injuries in both in vitro and in vivo systems. The WP depicted in the cartoon is a representative of WP components that possess disulfide bonds in their structure, such as albumin.

In this study, we converted WP into a potent thiol antioxidant through reductive modification. The converted WP exhibited strong antioxidative effects in both in vivo and in vitro models. The main findings are summarized in the schematical depiction in Figure 8.

Treatment of WP with reductive chemical DTT significantly increased the number and activity of -SH groups. This is because DTT can cleave the disulfide bonds in the protein structure [11] and expose free -SH groups. Recently, we have reported that the treatment of albumin with DTT, which has only one free SH group at cysteine 34 (Cys34), resulted in the appearance of more than 10 -SH groups through the splitting of intra-chain disulfide bonds and significantly enhanced antioxidative activity [11]. DTT treatment also split disulfide bonds in WP. This is supported by the fact that DTT treatment caused the shift of several protein bands from high MW to lower MW in SDS PAGE.

Using R-WP, we demonstrated that it displayed strong antioxidative activity at the molecular, cellular, and animal levels. The reductively modified protein significantly prevented H_2_O_2_-induced protein oxidation and cell injury. The potential mechanisms involved could be multiple. First, R-WP could directly scavenge H_2_O_2_ through a thiol-mediated reaction. Incubation of H_2_O_2_ with R-WP led to a significant reduction in H_2_O_2_ concentration. Second, -SH groups are especially susceptible to the oxidative modification of H_2_O_2_ and hydroxyl radicals [3,14,26,27]. In the presence of R-WP, H_2_O_2_ attacked the -SH groups in WP, instead of other structurally and functionally important cellular proteins, thus sparing cells from oxidative damage. Third, we demonstrated that the -SH groups in WP interacted with other small thiol antioxidant GSH and prooxidant GSSG through -SH/disulfide bond exchange. This reaction integrated R-WP into the thiol redox regulatory system, thus participating in thiol homeostasis [28,29]. Fourth, in vivo administration of WP may provide amino acids required for synthesizing other antioxidants, such as GSH [30], thus indirectly enhancing cellular antioxidative defense. Lastly, WP also has -SH-group-independent antioxidative actions [13,31,32,33], such as scavenging radicals and binding to metals [34,35], which could also contribute to the antioxidative effects.

We also observed that R-WP potently protected against renal cell injury in a mouse model of IRI. We used this model because the central role of ROS in this model has been well established [16,17]. The ROS from the renal infiltrated inflammatory cells and local cells initiates and exaggerates renal inflammation and injury. Antioxidative therapies have been shown to be effective in the prevention and treatment of renal IRI [36]. In consistency with previous reports, we demonstrated that the loss of renal function was associated with a marked increase in renal oxidation, inflammation, and cell injury. In addition, we provided the first evidence showing that the oral administration of dietary protein could also potentially prevent I/R renal damage via thiol-mediated antioxidative actions.

Intriguingly, we noticed that the IRI was associated with oxidative changes in the serum and colon, which could also be completely prevented by R-WP. This observation suggested that the renal oxidative stress could be transmitted to other organs via blood flow, in which ROS and ROS-oxidized proteins could be critically involved. The complete recovery of local and systemic OS by R-WP suggested that its protective actions may be achieved through rebalancing the whole-body redox state.

In this investigation, we chose H_2_O_2_ as a representative oxidant to test the effects of R-WP. The rationale for this selection was that H_2_O_2_ is particularly important among various ROS. It is generated through the SOD-catalyzed dismutation of superoxide, the predominant ROS produced at disease lesions [37,38]. It is the precursor of several highly reactive toxic radicals, such as peroxyl and hydroxyl radicals. Moreover, unlike other radicals, H_2_O_2_ can pass through the membrane, transmitting free radical-induced damage across cell compartments and between cells [39,40]. In serum and body fluid, detoxification of H_2_O_2_ and its hydroxyl radical products is predominantly performed by thiol antioxidant albumin [4,41,42,43]. Against this background, H_2_O_2_ could be the most likely oxidant targeted by R-WP.

Of note, H_2_O_2_ also plays a vital role in renal IRI. The infiltrated inflammatory cells and local resident cells have been shown to produce a large amount of superoxide and H_2_O_2_ [16,17,44,45]. Furthermore, treatment of I/R renal injury with catalase (the enzyme responsible for H_2_O_2_ decomposition) or H_2_O_2_-scavenging thiol antioxidant has been reported to be able to attenuate renal injury [46,47]. The current study provided additional evidence supporting the critical involvement of H_2_O_2_ in renal IRI, as shown by the fact that IRI was associated with a marked increase in local and systemic -SOH levels, which was suppressed by the thiol protein antioxidant. This is because -SOH is the product of the reaction between the protein cysteine thiol and H_2_O_2_ [48], the level of which indirectly reflects the level of H_2_O_2_ in vivo.

It is also worth mentioning that the antioxidative actions of R-WP on H_2_O_2_-induced cytotoxicity in culture and renal IRI were mediated by its -SH groups because the observed effects completely disappeared after the blockade of -SH groups with maleimide. WP has also been reported to exert antioxidative actions via alternative mechanisms, such as elevating intracellular GSH and binding to metals [30,31,34,35]. However, in the current investigation, the control WP only exerted a marginal effect. The reason for the discrepancy is currently unclear. It could be related to the different models and indicators used for the investigation. More detailed analysis is required in the future. One limit of the current study is that although we confirmed that Alb in WP may contribute to the observed effects, the main components involved remain to be characterized. One of the most interesting candidates could be β-lactoglobulin, which occupies 35% of WP and has two free thiols and four disulfide bonds in its structure [49,50]. Identifying and characterizing the main proteins involved could help to develop more effective dietary antioxidants.

It should also be mentioned that the components and functions of WPs vary with the sources of raw milk, their production procedures, and the methods used for sterilization. The WP used in the current investigation was sterilized through low-temperature pasteurization. This procedure caused less protein denaturation and maintained more bioactive proteins/peptides, compared to the standard higher-temperature procedure. Of note, the same WPs have been reported to have higher antibodies against pathogenic enteric microbes and exhibited a better protective effect on intestinal integrity [19,20,21].

Our study could have important implications. First, compared to currently available antioxidants, such as catalase, SOD, GSH, and NAC, R-WP has several advantages. It is safe, cheap, readily available, and has been extensively used as a protein supplement in daily life. Second, the results from the animal experiments provide additional evidence supporting the integration of the local and systemic oxidative status and challenging the traditional view that only emphasizes the importance of the intracellular compartment in cellular protection against oxidants. Third, WPs contain various proteins and peptides with versatile biological functions [12,13]. Previous studies have shown that bioactive peptides, released from the native proteins during gastric digestion, have anti-inflammatory, antibacterial, anti-cancer, and other health-promoting actions [12,13]. WPs have been used in the treatment of IBD, intestinal cancer, and rheumatic diseases [21,51,52,53]. Given that all these diseases are associated with oxidative stress [11,54,55], it is conceivable that the R-WP protein could have a wide range of applications.

## 5. Conclusions

In conclusion, we have successfully converted WP as a potent dietary thiol antioxidant and demonstrated that it could be used to rebalance the systemic redox state and to treat oxidative-stress-centered renal diseases. As a next step, we will characterize the components involved in the antioxidative actions and verify the effectiveness of R-WP in the prevention and treatment of other diseases. Given that oxidative stress is a common mechanism behind various conditions, such as cardiovascular diseases, cancer, and aging, and that R-WP has advantages over other available antioxidants in terms of safety, cost, nutrient value, and multiple biological activities, R-WP could have wide applications. 

## Data Availability

The data are contained within the article.
